# Experimental peri-implantitis induces neuroinflammation: An exploratory study in rats

**DOI:** 10.1186/s12903-024-04995-z

**Published:** 2024-10-18

**Authors:** Emilio A. Cafferata, Ausra Ramanauskaite, Astrid Cuypers, Karina Obreja, Eva Dohle, Shahram Ghanaati, Frank Schwarz

**Affiliations:** 1 Department of Oral Surgery and Implantology, Goethe University, Carolinum, Frankfurt am Main, Germany; 2 Department of Periodontology, School of Dentistry, Universidad Científica del Sur, Lima, Perú; 3 Frankfurt Oral Regenerative Medicine (FORM-Lab), Clinic for Maxillofacial and Plastic Surgery, Goethe University, Frankfurt am Main, Germany

**Keywords:** Peri-implantitis, Rat, Animal model, Neuroinflammation, Neurodegeneration, Neurodegenerative disease, Alzheimer’s disease

## Abstract

**Purpose:**

Cumulating evidence supports the close association between periodontal diseases, neuroinflammation and neurodegenerative pathologies, except for peri-implantitis (PI). Thus, this study explored the association between experimental PI and neuropathological changes in the rat brain.

**Materials and methods:**

After bilateral first molars extraction, experimental PI was induced at titanium implants placed in the maxillae by lipopolysaccharide injections and ligature placement. Following 28-weeks of disease progression, the maxillae and brains were retrieved from 6 rats. Healthy brains from 3 rats were used as control. Brains were analyzed by immunohistochemistry to detect signs of neuroinflammation (interleukin (IL)-6 and tumor necrosis factor (TNF)-α)), microglial activation (IBA-1) and astrogliosis (GFAP). To explore signs of neurodegeneration, hematoxylin/eosin and Nissl stainings were used. Also, four different antibodies against amyloid beta (Aβ 1–42) were tested.

**Results:**

Chronic PI lesions showed peri-implant bone resorption accompanied by large inflammatory infiltrates. IL-6^+^ and TNF-α^+^ cells were found within the CA1 and Dentate Gyrus regions of the hippocampus of the PI-affected group, while almost no immune-positivity was detected in the control (*p* < 0.05). Detection of activated GFAP^+^ microglia and IBA-1^+^ astrocytes surface were significantly higher at the CA areas, and cerebral cortex of the PI-affected group, in comparison with control (*p* < 0.05). Shrunk neurons with pyknotic nuclei were inconsistently found among the PI-affected group, and these were almost not detected in control. No positive Aβ reactivity was detected in any of the samples.

**Conclusion:**

Chronic experimental PI lesions led to an increased detection of IL-6 and TNF-α, GFAP^+^ microgliosis and IBA-1^+^ astrocytosis, and in some cases, neurodegeneration, in the rat brain.

## Background

Peri-implantitis (PI) is an etiologically complex oral inflammatory disease that occurs around dental implants, affecting every fifth patient bearing implant-supported prostheses [1]. During this pathological condition, it is presumed that dysbiosis within the peri-implant microbiota leads to chronic inflammation and progressive loss of supporting bone, which ultimately leads to implant loss [2–4]. In a similar manner, periodontitis arises from a subgingival dysbiotic microbiota that provokes an osteolytic immune response that leads to tooth loss, affecting almost half of the American adult population [5]. Apart from its dysbiotic origin, PI lesions also share many similarities with those noted at periodontitis sites, such as the presence of macrophages, plasma cells and T lymphocytes, though in larger proportions [6, 7]. In this context, similar levels of T helper type (Th) 1 and Th2 cells, among other infiltrating inflammatory cells, have been found in experimental PI and periodontitis lesions [8]. Nonetheless, significant differences among microRNAs expression, regulating T- and B- cell receptor signaling, between PI and periodontitis experimental lesions have also been detected [9], suggesting the involvement of different inflammation regulation pathways behind the onset of the diseases. Indeed, a significantly increased expression of nuclear factor κB and matrix metalloproteinase 8 found in PI lesions, in comparison with periodontitis lesions, also suggests a more pronounced inflammation-driven tissue destruction during PI [10]. Consequently, PI lesions present highly differing patterns of bone loss, frequently showing greater amounts of bone loss, less trabecular density and increased number of osteoclasts, in comparison with experimental periodontitis lesions [10]. Therefore, it can be suggested that even though PI and periodontitis share many features, regarding their origin and onset, the inflammatory pathways that lead to the development of their respective heterogenous chronic lesions may be substantially different.

The microbial profiles associated with PI and periodontitis are similar regarding the incidence and relative abundance of particular periodontopathic bacteria, such as *Porphyromonas gingivalis* and *Prevotella intermedia*/*nigrescens*, capable of promoting dysbiosis and deregulating the local immune response [11–13]. Consequently, the constant bacterial challenge and subsequent chronic inflammation promote the release of bacterial products and pro-inflammatory mediators into systemic circulation. Indeed, numerous clinical studies have shown that local inflammatory lesions, such as periodontitis and PI, may also induce transient bacteremia and low-grade systemic inflammation [14–17]. In this context, periodontitis, arising from microbial dysbiosis, has been strongly associated to neuroinflammation, cognitive decline, and the presence of histopathological signs compatible with neurodegenerative disorders, like Alzheimer’s disease (AD), such as reactive gliosis and amyloid β (Aβ) peptide accumulation [18–20]. In fact, periodontitis is commonly detected among patients affected by neuroinflammation-driven diseases [21], being diagnosed more frequently between AD patients compared to controls (20.7% versus 6.7%) [22], and Parkinson’s disease (PD)-affected patients frequently present more severe clinical parameters of periodontitis [21].

Furthermore, the presence of periodontopathogens, such as *P. gingivalis* and *Treponema denticola*, as well as lipopolysaccharide (LPS) and gingipain from *P. gingivalis*, have been detected in the post-mortem brain tissue or serum of individuals affected by neurodegenerative disorders, such as AD and PD [23, 24]. Based on the findings of a recent preclinical study, the repeated oral application of *P. gingivalis* led to neurodegeneration and the production of Aβ 1–42 in the brain tissues of mice [25]. In consequence, different hypotheses have been postulated to shed light into these associations, mainly suggesting the existence of a dysbiosis/inflammation-driven oral-brain or oral-gut-brain axis that can lead to neurodegenerative changes in the brain [26]. Among these, constant bacteremia, provoked by periodontitis or even chewing, can induce low-grade inflammation and the translocation of pro-inflammatory cytokines through the blood-brain barrier [27, 28], which leads to reactive astrocytes producing interleukin (IL)-6 and tumor necrosis factor (TNF)-α, and microglia polarizing towards a neuroinflammatory M1 phenotype [29, 30]. Otherwise, some studies have also postulated that oral bacteria, such as *T. denticola*, may enter the brain through the peripheral endings of the trigeminal nerve [31]. Apart from that, rising evidence suggests that oral dysbiosis may trigger gut microbiota dysbiosis [26, 32]. For instance, the oral pathobiont, *P. gingivalis*, is capable of directly inducing metabolic changes associated with alteration and dysbiosis of the gut microbiota and, at the same time, significantly impair the intestinal barrier function which leads to bacteremia and systemic inflammation [33, 34]. Altogether, these insults compromise the intestinal barrier permeability and induce an intestinal immune response that is capable of provoking neuroinflammation [26, 32].

Our group has consistently used a modified version of the PI-model proposed by Takamori. et al. [35–37], using the combination of LPS and ligature, a highly immunogenic stimuli and proven neurotoxin, and an oral bacterial-cumulating artifact, aiming to emulate the dysbiotic etiology of the disease and the establishment of a true chronic lesion. Since the origin of periodontal and peri-implant lesions share many similarities [6, 7], it might be hypothesized that chronic inflammation at tissues surrounding dental implants may also be linked to neuroinflammation in the brain. Therefore, the aim of this pilot study was to investigate the possible association between experimentally induced PI lesions and neuropathological changes in the brain of rats.

## Methods

### Animals

Nine male Wistar rats (age: 10 months, mean weight 725 ± 88 g) were used in the present study. For this initial experimental exploratory study, six animals were assigned to the experimental peri-implantitis (PI) group (which belonged to a previous animal cohort [35]), and three healthy rat brains (without intervention), obtained separately, were used as the control group. All animals were individually housed in a controlled environment, under standard conditions of temperature, light, and ventilation. Animals were provided with water and food pellets *ad libitum*. The study protocol considered the 3Rs (Replace, Reduce, Refine) guideline for animal experimentation and was approved by the appropriate local authority (Regierungspräsidium Darmstadt, Germany). Subsequently, the reporting of the study adhered to the ARRIVE Guidelines [38].

### Anesthesia protocol

For each surgical intervention, the animals were anesthetized by intraperitoneal injection of 7.5 mg/kg ketamine (Ketanest^®^, Pfizer Pharma GmbH, Karlsruhe, Germany) and 5 mg/kg xylazine (Rompun^®^, Bayer HealthCare, Leverkusen, Germany). In order to ensure full anesthesia, animals were closely monitored until complete stop of movement and then tested for lack of righting reflex before the start of any procedure. For postoperative analgesia, 4.5 mg/kg carprofen was administered subcutaneously immediately after surgery, as well as 1, 2, and 3 days postoperatively.

### Induction of peri-implantitis

**Fig. 1:**
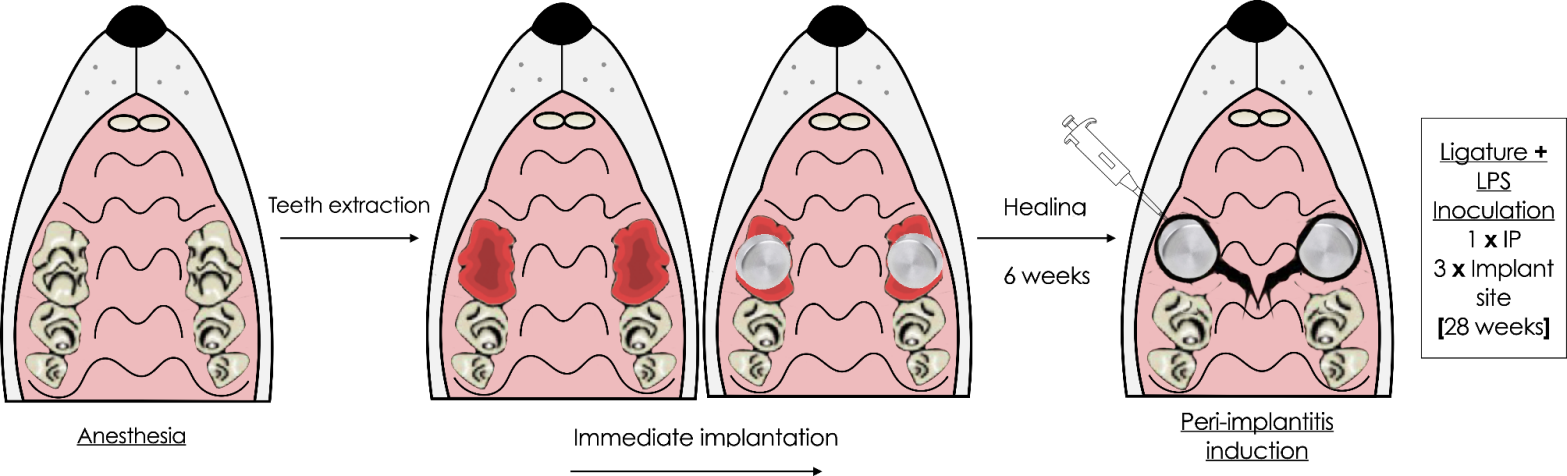
**Rat chronic peri-implantitis model**. Diagram showing procedures flow to experimentally induce peri-implantitis. Under general anesthesia, bilateral maxillary 1st molars were extracted, followed by immediate implantation. After 6 weeks of healing, peri-implantitis was induced by the combination of one LPS IP injection, one daily local application of LPS for 3 days and ligature placement. Peri-implantitis chronic progression lasted 28 weeks. LPS: lipopolysaccharide; IP: intraperitoneal

Following the extraction of both maxillary first molars, smooth-surfaced titanium mini-implants (Ustomed^®^ Micro-Screws, Cross, ⌀ 1.2 mm, shortened to 3 mm) were immediately inserted into the alveolar sockets and left to heal for 6 weeks [36]. Subsequently, peri-implantitis lesions were induced by following a stablished and validated procedure [37]. For this, all animals belonging to the PI group were first immunized with an intraperitoneal booster injection of 150 µg of *Escherichia coli* LPS (O111:B4, EMD Millipore, Merck, Darmstadt, Germany) in 0.3 ml of PBS, followed by daily topical 50 µg LPS applications in the peri-implant sulcus at each implant site for 3 days. As previously reported, pre-immunization favors the accumulation of larger immune cells infiltrate and bone resorption area in peri-implant tissues than in non-immunized animals, presumably due to the formation of specific antibody-antigen complexes after antigen infiltration in peri-implant tissues, which further promotes chronic inflammation [37]. Subsequently, miniature polyester ligatures (7 − 0) were placed in a submarginal position around implants for 4 weeks. In brief, ligatures were forced into a position directly apical to the mucosal margin and a “pocket” was created to facilitate the microbial colonization, as already reported by our group [35] (Fig. 1). After 12 weeks of peri-implantitis induction, a surgical surface decontamination of the peri-implantitis affected implants was conducted to reduce the risk of implant loss. This was followed by another healing period of 12 weeks. Accordingly, the observation period amounted to 28 weeks following the induction of peri-implantitis lesions. Experimental PI was considered as successfully established, if bone resorption and migration of junctional epithelium beyond the first implant thread, and an inflammatory infiltrate were present at the PI lesion by means of hematoxylin/eosin staining [37]. For the control group, healthy animals receiving no intervention were used.

### Samples recovery and histological preparation

Animals were euthanized by using an overdose of pentobarbitone at 100 mg/kg, while the tissues were fixed by means of transcardial perfusion with 10% buffered formalin. Afterwards, brains and maxillae samples were immediately retrieved. Brains were post-fixated in 10% buffered formalin for 24 h. Maxillae were first demineralized with 4% EDTA for 30 days. Then, all specimens, were dehydrated using ascending grades of alcohol and xylol, and embedded in paraffin for conventional hematoxylin/eosin, Nissl staining, or immunohistochemical analyses.

### Immunohistochemistry

After deparaffinization, rehydration, and mounting of 3 μm-thin coronal tissue sections on positively charged glass slides, antigen unmasking was performed by heating the samples for 30 min in a buffer citrate solution at 96 °C. After quenching the activity of endogenous peroxidase with hydrogen peroxide block for 10 min at room temperature, non-specific binding sites were blocked with Ultra V Block solution for 5 min (Ultravision Quanto Detection System HRP, Thermofisher).

Then, the tissue sections were incubated with the primary antibodies against IL-6 (clone 1.2-2B11-2G10) (dilution: 1:100, Abcam), TNF-α (dilution: 1:400, Invitrogen), IBA-1 (dilution: 1:400, Invitrogen), or GFAP (clone GA5) (dilution: 1:400; Invitrogen). Apart from that, the following monoclonal antibodies against Aβ were applied: (1) rabbit (H31L21) (dilution: 1:500; Invitrogen), (2) human (1–42) (dilution: 1:500; Bioscience), (3) mouse IgG1 (1–16 clone 6E10) (dilution 1:500; BioLegend), and (4) Anti-Beta Amyloid (17–24; clone 4G8) (dilution: 1:500; Covance).

For the visualization of IL-6, TNF-α, IBA-1 and GFAP detection: After incubating for 1 h for IL-6, IBA-1 or GFAP and 0.5 h for TNF-α in humid chambers at room temperature, the Ultravision Quanto Detection System HRP (Thermofisher) manufacturer’s protocol was followed. AEC was used as chromogen for IL-6 and DAB, for TNF-α, IBA-1 and GFAP. Then, sections were counterstained with Mayer’s hematoxylin. As negative controls, unspecific antibodies (rabbit IgG or mouse IgG_1_) and the omission of primary antibodies were applied.

For the visualization of Aβ detection: The slides were washed in PBS and incubated with secondary biotinylated anti-mouse antibodies (dilution: 1:50, Dako) for 90 min at room temperature. After washing in PBS, the presence of antibody-antigen complexes was visualized using a streptavidin-peroxidase solution (dilution: 1:250, Dako) and AEC (3-amino-9-ethylcarbazole) as the chromogen (Dako). An unspecific antibody (mouse IgG_1_) (Dako) served as negative control.

### Immunohistochemical analysis

Digital images (original magnification x 200; BX53, Olympus, Hamburg, Germany) were obtained from each specimen and evaluated using the cellSens or ImageJ software. For the IBA-1 and GFAP percentage coverage analysis, images were converted to 8-bit format and binary thresholds were minimally adjusted before background subtraction for the best representation of the raw data. Artifacts were manually and individually filtered out before the calculation of the area covered by dark (positive) pixels, as previously described [39]. In addition, a skeletal analysis was performed for the IBA-1 stained slides using the ImageJ skeletonize plugin. Briefly, binarized images were produced by 8-bit conversion and contrast/threshold adjustment to adequately visualize microglia branches. “Despeckle”, “close” and “remove outliers” functions were applied to remove noise, and images were skeletonized. Microglial branches length was calculated across the images and averaged by the number of cells per image, as previously described [39].

In all sections, the frontal, parietal and temporal cortex as well as the hippocampus were defined as regions of interest (ROI). Within the ROIs, the presence IL-6, TNF-α, IBA-1 or GFAP positive cells/surface were described.

### Statistics

For the relative quantification of IL-6, and TNF-α positive cells, or IBA-1 and GFAP positive covered area by immunohistochemistry, ten random slides from each sample were chosen, and positive cells/surface percentage present in the cerebral cortex or hippocampus ROIs were calculated, and expressed as mean and standard deviation. Data were statistically analyzed using the Jamovi v.2.3 software. Due to the exploratory nature of the study, statistical differences were determined using the non-parametric Mann-Whitney U test. The level of significance was set at *p* < 0.05.

## Results

### Experimental peri-implantitis model

**Fig. 2:**
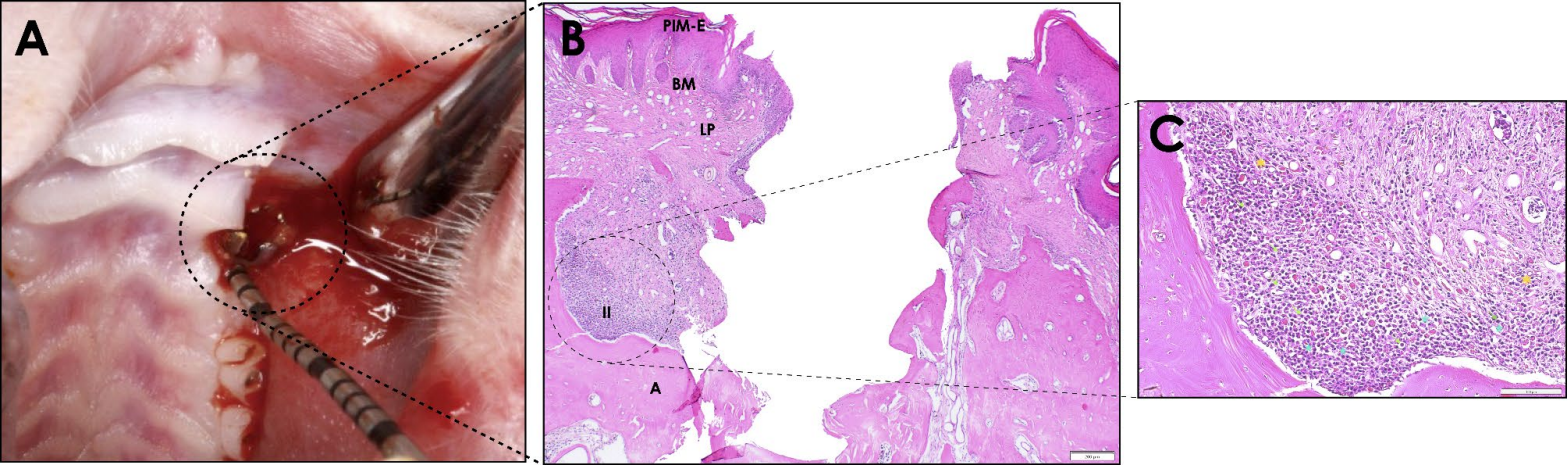
**Experimental peri-implantitis**. Chronic-type peri-implant lesions were induced at titanium implants placed in the upper jaw of rats. **(A)** Clinical signs of inflammation were evidenced by the presence of bleeding on probing and suppuration at ligature removal. **(B)** Representative image showing haematoxylin & eosin staining of an experimental peri-implantitis lesion [10x]. **(C)** [20x] augmentation on **B** image showing PI lesion inflammatory infiltrate, with the presence of polymorphonuclears, monocytes and some lymphocytes. PIM-E: peri-implant mucosa epithelium; BM: basal membrane; LP: lamina propia; II: inflammatory infiltrate; AB: alveolar bone; ↘ polymorphonuclear cell; ★: monocyte cell; ✶: lymphocyte cell

Evident signs of alveolar bone resorption and apical migration of peri-implant tissues were compatible with a successful induction of experimental PI after 28 weeks (Fig. 2). Local edema and bleeding, clinical signs of peri-implant inflammation, can be appreciated next to the ligature-tied implants, as shown in Fig. 2A. Histologically, a dense infiltration area of inflammatory cells below the remains of the peri-implant mucosa epithelium and the connective tissue can be observed (Fig. 2B), enriched in polymorphonuclear and monocytic cells, and few lymphocytes (Fig. 2C).

### Hippocampus and cerebral cortex morphological characteristics

**Fig. 3:**
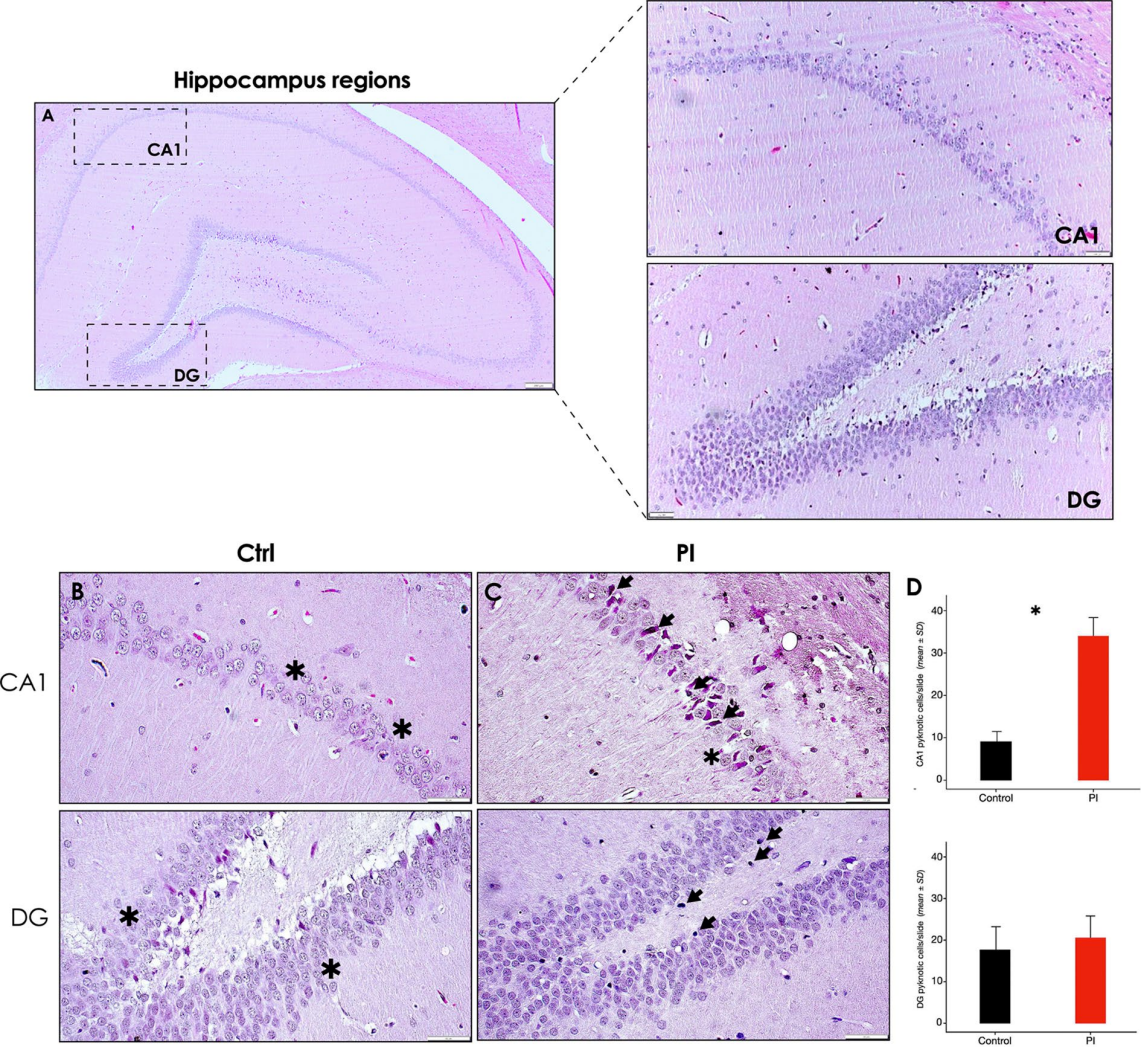
**Hippocampus H&E staining showing cellular morphological alterations following experimental PI**. **A)** Representative image showing the hippocampus regions -CA1, CA2, C3, CA4 and DG-, the regions of interest CA1 and DG are delimited by dashed squares [5x]; Right: Representative images of the CA1 (superior) and DG (inferior) hippocampus regions of the control group [10x]. **B)** and **C)** Representative images of the CA1 (superior) and DG (inferior) hippocampus regions of the control and peri-implantitis group, showing morphologically altered neurons and glial cells [40x]. **D)** Relative quantification of pyknotic cells in the CA1 and DG regions of the hippocampus of rats in control and PI groups. Data are represented as mean ± SD pyknotic cells from 10 random slides per sample. *p < 0.05.CA: Cornu ammonis; DG: dentate gyrus; PI: peri-implantitis group; CTRL: control group; ↘: pyknotic pyramidal neuron; *: normal neuron. Figure scales correspond to: superior [200 μm], superior right [100 μm] and inferior [50 μm] figures

Significantly more, deeply stained and morphologically altered – condensed, with pyknotic nuclei and uneven cytoplasm distribution –, and irregularly distributed pyramidal neurons (Fig. 3C – superior [arrow]) can be seen in the hippocampus Cornu ammonis (CA)1 region of the peri-implantitis affected group slides in comparison with the control group (Fig. 3B) (*p* < 0.05) (Fig. 3D), which could be signs associated to neurodegeneration and neuroinflammation. Apart from that, the Dentate Gyrus (DG) region also presents cells with condensed nuclei mainly distributed along the subgranular zone, below and adjacent to the granular cell zone, in some sections of the hippocampus of the PI group in comparison to the control group, although no significant differences were found between groups (*p* > 0.05) (Fig. 3D).

**Fig. 4:**
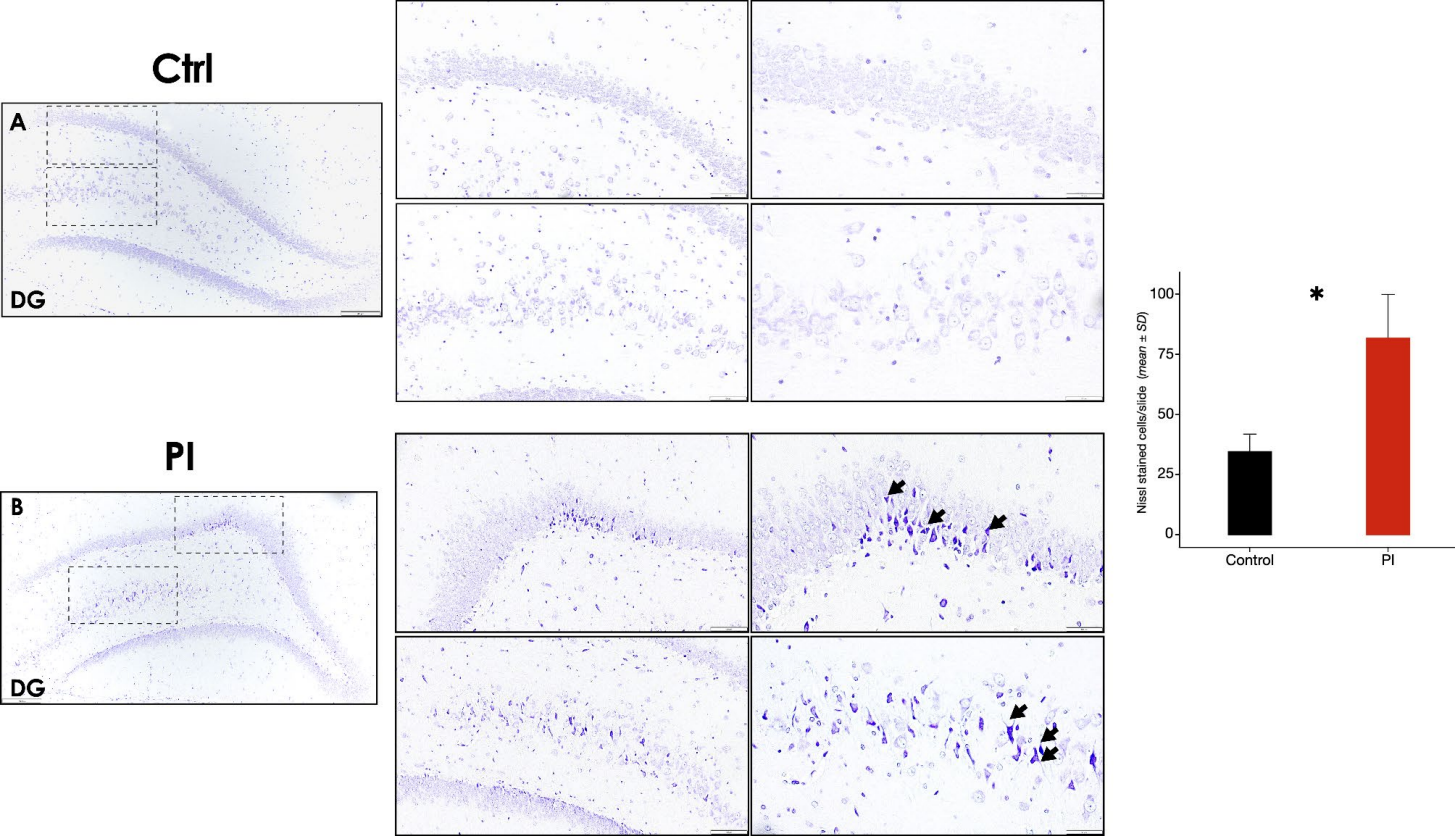
**Hippocampus Nissl staining showing cellular morphological alterations following experimental PI**. Representative images showing the DG region from the control **A)** and PI **B)** groups, zoom augmented areas are delimited by dashed squares [10x]. Control middle [20x] and right [40x] panels. PI middle [20x] and right [40x] panels. **C)** Relative quantification of Nissl-stained cells in the DG region of the hippocampus of rats in control and PI groups. Zoomed areas are delimited by grey dotted lines. Data are represented as mean ± SD deeply stained cells (hyperchromatic) from 10 random slides per sample. *p < 0.05. DG: dentate gyrus; PI: peri-implantitis group; CTRL: control group; ↘: hyperchromatic cell. Figure scales correspond to: left [200 μm], middle [100 μm] and right [50 μm] figures

Complementarily, Nissl staining showed similar patterns of marked neuronal staining, frequently showing hyperchromatic cells with darkly stained and condensed nuclei unevenly distributed among the DG region of the hippocampi of the PI-affected group slides, in comparison with the control group, which showed lighter or unevenly stained cells among the same hippocampus regions (Fig. 4). Neurons in apparent degenerative state, shown as shrunken and deeply stained cells (Fig. 4B [arrow]), were significantly more frequently detected in the DG region of the PI-affected group samples, in comparison with the control group (*p* < 0.05) (Fig. 4C).

### IL-6 and TNF-α hippocampus immunostaining

**Fig. 5:**
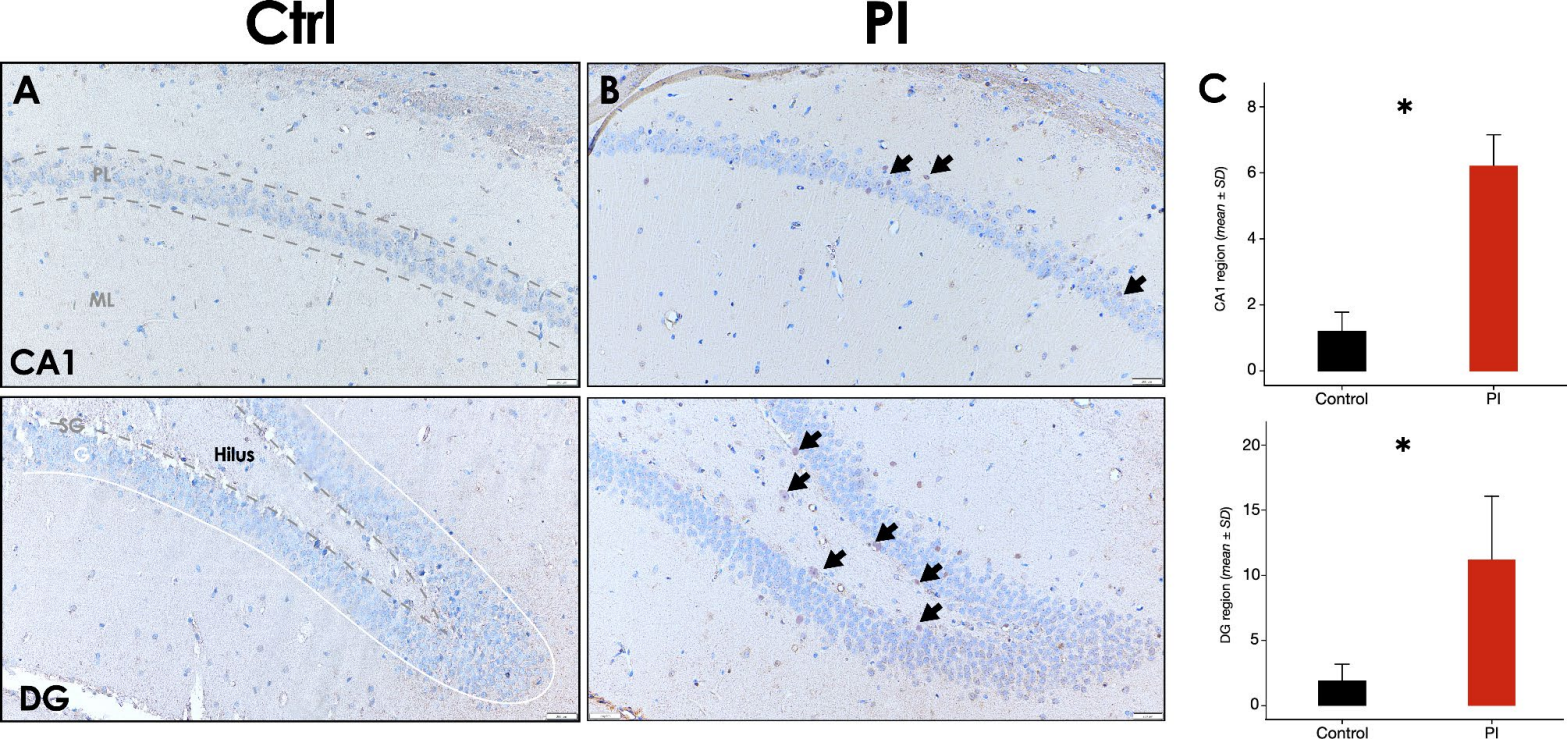
**Hippocampus IL-6 immunostaining following experimental PI**. **A)** Representative hippocampus image of the control group, showing the CA1 (superior) and DG (inferior) regions [10x]. ** B)** Representative hippocampus image of the peri-implantitis group, showing the CA1 (superior) and DG (inferior) regions [10x]. **C)** Relative quantification of IL-6^+^ cells in the CA1 and DG regions of the hippocampus of rats in control and PI groups. PL and SG zones are delimited by grey dotted lines, the zone G is delimited between the grey dotted line and the continuous white line, and the Hilus zone is comprehended between the SG zone and the CA4 region. Data are represented as mean ± SD positive cells from 10 random slides per sample. *p < 0.05. PL: pyramidal layer; ML: molecular layer; SG: subgranular cell zone; G: granular cell zone; ↘: IL-6 positive cell. Figure scales correspond to: left [200 μm], and right [100 μm] figures

Intracellular positive immunodetection of IL-6 can be observed in few cells in the pyramidal cell layer among the CA1 region (Fig. 5B – superior [arrow]) of the hippocampus of most of the peri-implantitis affected group slides, otherwise almost no positive IL-6 immunodetection can be observed in the healthy group slides (Fig. 5A – Superior). Similarly, few IL-6 expressing cells can only be observed in the DG region, in the subgranular and hilus zones (Fig. 5B – inferior [arrow]), of the peri-implantitis affected group (Fig. 5). In fact, significantly more IL-6 positive cells were observed in the CA1 and DG regions of the hippocampus in the PI group, in comparison with the healthy group (*p* < 0.05) (Fig. 5C).

**Fig. 6:**
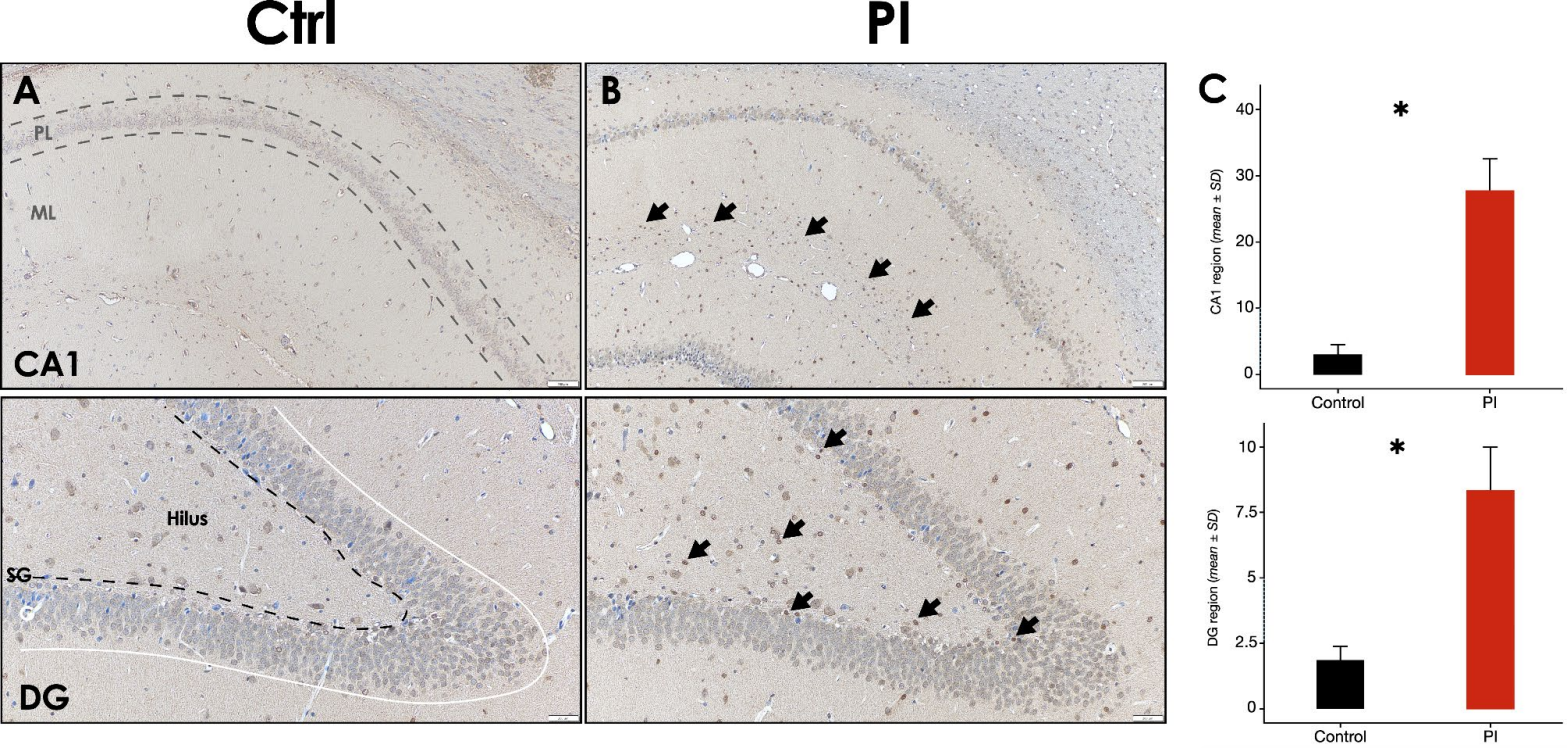
**Hippocampus TNF-α immunostaining following experimental PI**. **A)** Representative hippocampus image of the control group, showing the CA1 (superior) and DG (inferior) regions [10x]. **B)** Representative hippocampus image of the peri-implantitis group, showing the CA1 (superior) and DG (inferior) regions [10x]. **C)** Relative quantification of TNF-α^+^ cells in the CA1 and DG regions of the hippocampus of rats in control and PI groups. PL and SG zones are delimited by dark grey dotted lines, the zone G is delimited between the dark grey dotted line and the continuous white line, and the Hilus zone is comprehended between the SG zone and the CA4 region. Data are represented as mean ± SD positive cells from 10 random slides per sample. *p < 0.05. PL: pyramidal layer; ML: molecular layer; SG: subgranular cell zone; G: granular cell zone; ↘: TNF-α positive cell. Figure scales correspond to: left [200 μm], and right [100 μm] figures

In contrast, intracellular positive immunodetection of TNF-α was irregularly found between the hippocampus samples from the PI-group, and was rarely detected in the samples from the healthy group. TNF-α expression was detected in some of cells within the molecular layer of the CA1 region (Fig. 6B – superior [arrow]), and in the subgranular and hilus zones (Fig. 6B – inferior [arrow]) of the PI affected hippocampus (Fig. 6). Indeed, significantly more TNF-α positive cells were observed in the CA1 and DG regions of the hippocampus in the PI group, in comparison with the healthy group (*p* < 0.05) (Fig. 6C).

### IBA-1+ microglia and GFAP+ astrocytes detection

**Fig. 7:**
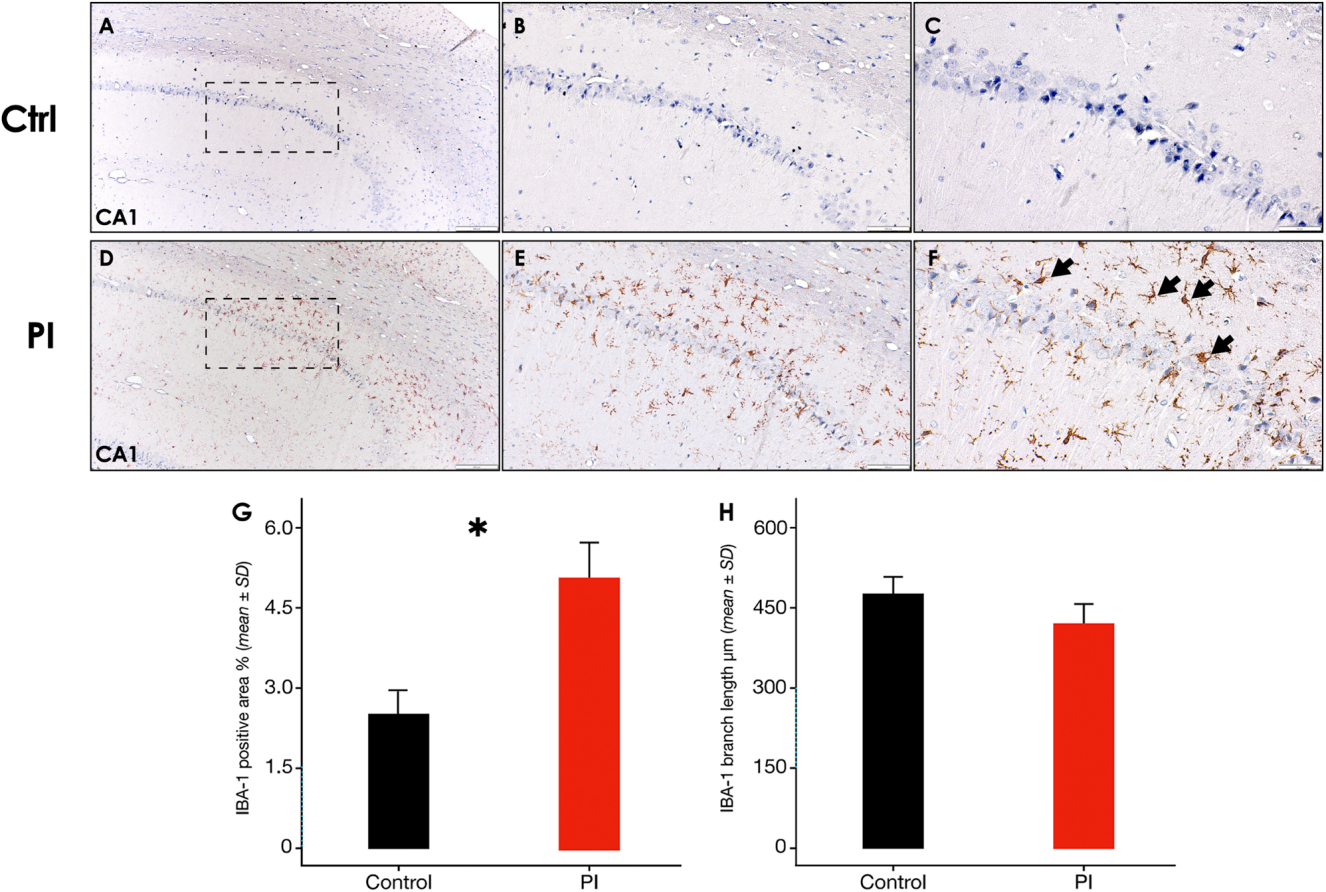
**Hippocampus IBA-1 immunostaining following experimental PI**. **A)** Representative hippocampus image of the control group, showing the CA1 region, a zoom augmented area is delimited by a dashed square [10x], **B)** [20x] and **C)** [40x]. **D)** Representative hippocampus image of the peri-implantitis group, showing the CA1 region, a zoom augmented area is delimited by a dashed square [10x], **E)** [20x] and **F)** [40x]. **G)** IBA-1^+^ surface coverage percentage and **H)** IBA-1^+^ Microglia branch length in the CA1 region of the hippocampus of rats in control and PI groups. Data are represented as mean ± SD of the percentage of positively stained area from 10 random slides per sample, or mean ± SD of the branches length divided by the number of cells per slide from 10 random slides per sample. *p < 0.05. PI: peri-implantitis group; CTRL: control group; ↘: IBA-1 positive cell. Figure scales correspond to: left [200 μm], middle [100 μm] and right [50 μm] figures

Activated IBA-1^+^ microglia were frequently detected and irregularly distributed mostly within the CA1 and CA2 regions of the hippocampus of the PI-affected group (Fig. 7D). In contrast, almost no IBA-1 positive cells were detected within the control group slides (Fig. 7A). IBA-1^+^ microglia in the PI-group showed irregular protrusions, and hypertrophic or bushy morphology (Fig. 7F [arrow]), compatible with microgliosis. Accordingly, IBA-1^+^ area was significantly higher in the PI-group, than the control (*p* < 0.05) (Fig. 7G). However, no statistically significant differences between groups were revealed by skeletal analysis (*p* > 0.05) (Fig. 7H).

**Fig. 8:**
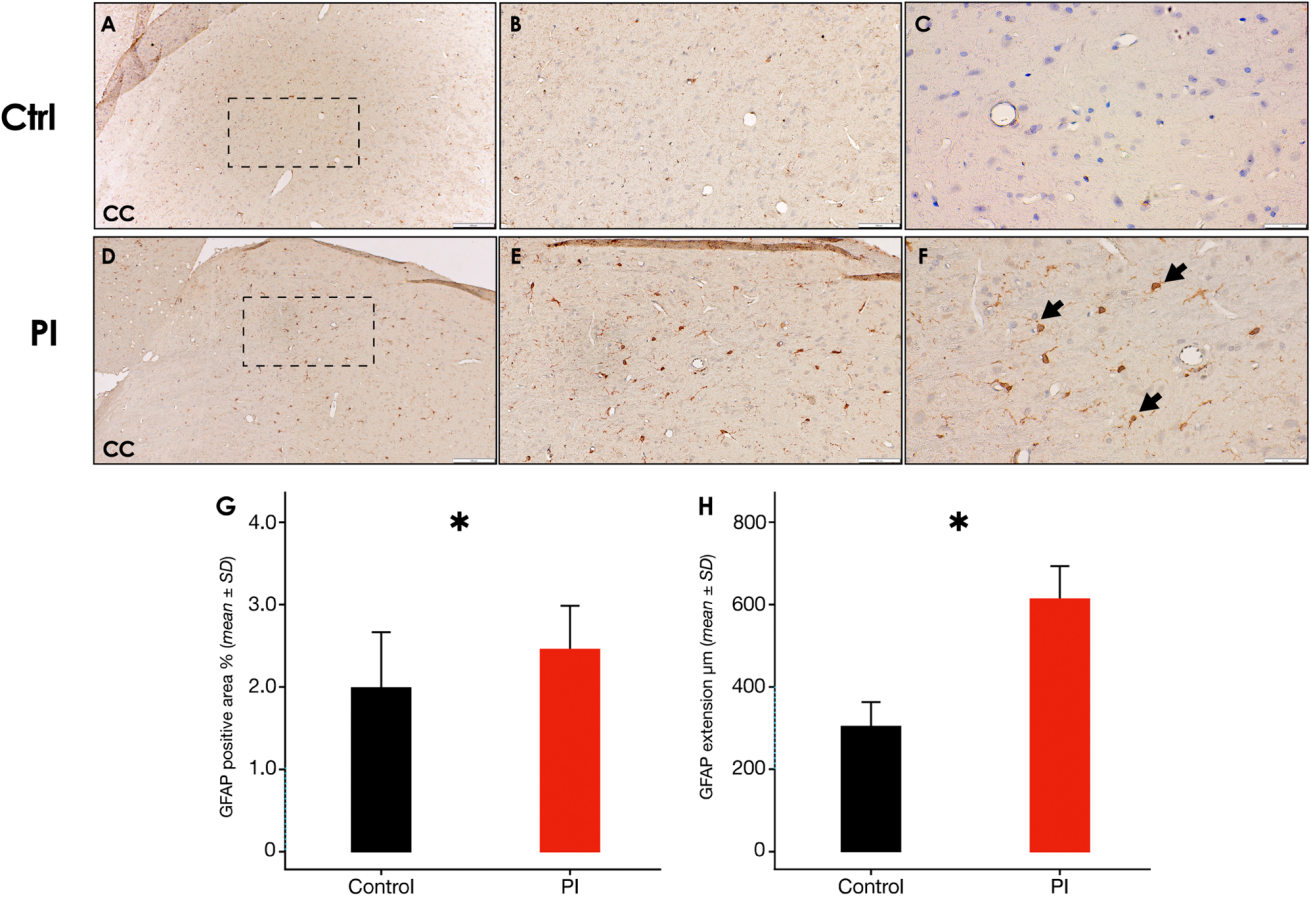
**Cerebral cortex GFAP immunostaining following experimental PI**. **A)** Representative image of the control group showing the cerebral cortex, a zoom augmented area is delimited by a dashed square [10x], **B)** [20x] and **C)** [40x]. **D)** Representative image of the peri-implantitis group showing the cerebral cortex, a zoom augmented area is delimited by a dashed square [10x], **E)** [20x] and **F)** [40x]. **G)** GFAP^+^ surface coverage percentage in the cerebral cortex of rats in control and PI groups. Data are represented as mean ± SD of the percentage of positively stained area from 10 random slides per sample. *p < 0.05. PI: peri-implantitis group; CTRL: control group; ↘: GFAP positive cell. Figure scales correspond to: left [200 μm], middle [100 μm] and right [50 μm] figures

Otherwise, reactive GFAP^+^ astrocytes were more frequently detected in the cerebral cortex of the brain samples of the PI-affected group, in comparison with the control group (Fig. 8F [arrow]). In contrast, GFAP^+^ cells were scarcely and irregularly detected within the DG regions of both the PI and control group samples. Nevertheless, GFAP^+^ area was significantly higher in the PI-group, than the control (*p* < 0.05) (Fig. 8G).

### Aβ detection

**Fig. 9:**
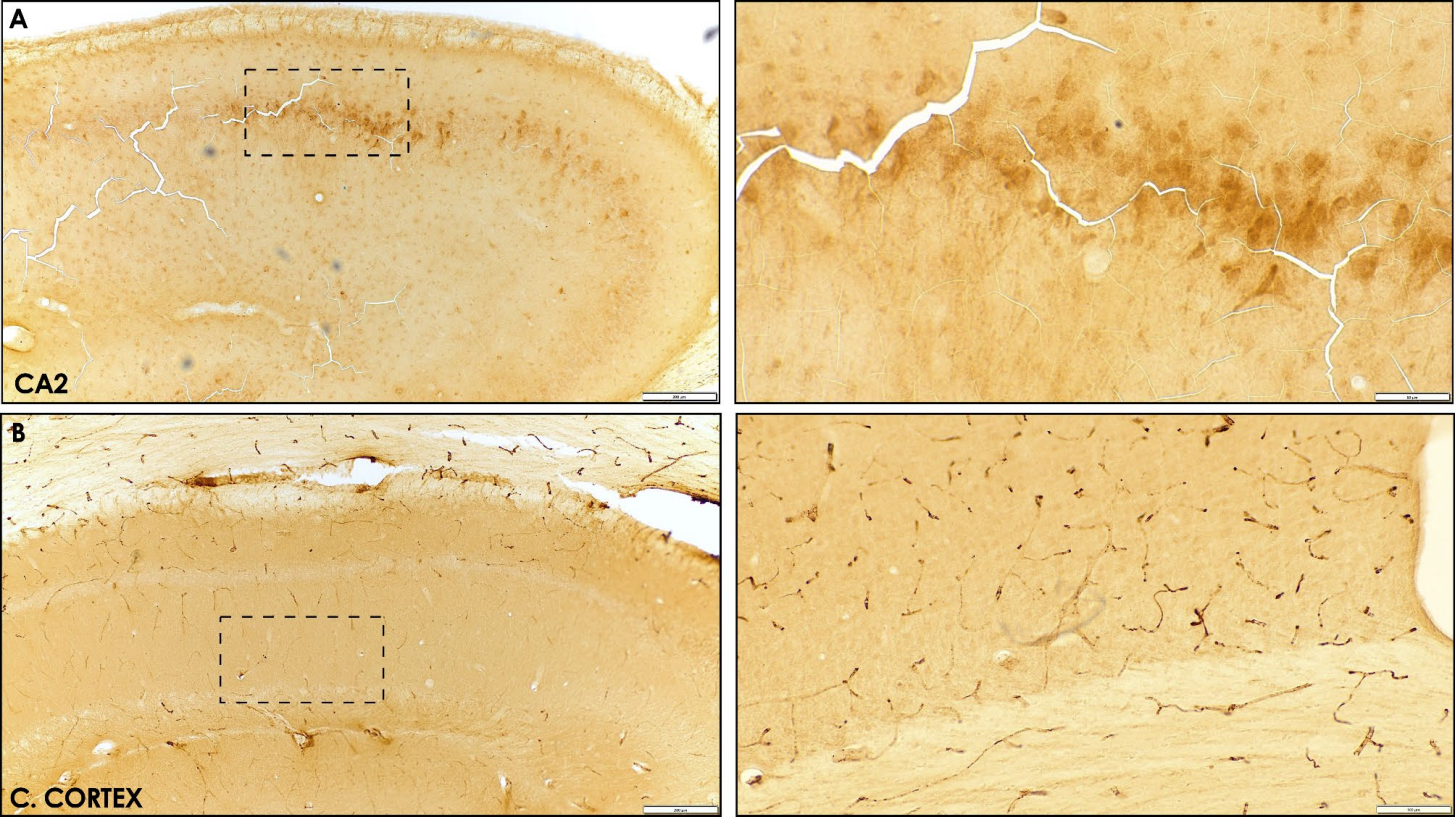
**Representative hippocampus and cerebral cortex Aβ 1–42 immunostaining following experimental PI**. **A)** CA2 region of the hyppocampus, a zoom augmented area is delimited by a dashed square [Right]. **B)** Cerebral cortex, a zoom augmented area is delimited by a dashed square [Right]. No Aβ 1–42 positive immunostaining could be detected in any of the specimens, either on PI or control group. Figure scales correspond to: superior Fig. [200 μm], inferior Fig. [100 μm]

Representative histological views of the ROI demarcated in different antibody groups are presented in Fig. 9. None of the specimens investigated revealed any signs of a positive Aβ antigen reactivity within the cerebral cortex or the hippocampus, as assessed by different antibodies.

## Discussion

Peri-implantitis, such as other periodontal diseases, has the potential to facilitate the infiltration of pro-inflammatory mediators into the bloodstream and further contribute to inflammatory disease development in distal organs [40]. Moreover, persistent, and chronic inflammation consistently takes place during neurodegenerative diseases, like AD and PD [41]. The present experimental study aimed to explore the possible association between experimentally induced PI and neuropathological changes in the brain of rats. On the basis of our immunohistochemical findings, experimental PI led to the detection of elevated levels of the pro-inflammatory cytokines IL-6 and TNF-α, and the markers IBA-1 and GFAP associated to activated microglia and astrocytes in the rat hippocampus and cerebral cortex, signs compatible with neuroinflammation associated to neurodegenerative diseases. However, neither extracellular nor intracellular Aβ 1–42 accumulation could be detected in any of the brain specimens. Altogether, these data suggest a possible link between chronic inflammation and/or dysbiosis occurring during peri-implantitis with neuroinflammation.

Recently, the possible link between oral dysbiosis/chronic inflammation, neuroinflammation and neurodegenerative disorders has been explored by using different oral dysbiosis animal models. Accordingly, a study using a ligature model of periodontitis, supporting oral local dysbiosis and inflammation, found increased levels of the pro-inflammatory mediators, IL-6, TNF-α, and Aβ 1–42 in the cerebral cortex, and IL-1 in the hippocampus, compatible with neurodegenerative signs of AD, in comparison with control rats [42]. In addition, when *P. gingivalis* or its LPS, a keystone pathogen for periodontal dysbiosis and one of its most inflammogenic virulence factors, were added to the ligature during experimental periodontitis, they led to a further increase of IL-6 levels, Aβ deposition and memory function impairment [43, 44]. Otherwise, the oral administration of *P. gingivalis* in leucine-rich repeat kinase (LRRK)2 mutant animals, for the emulation of late-onset PD, also led to an increase of the expression of TNF-α, IL-1β, IL-17 A and IL-17RA in the animals’ colon and brain, as well as degeneration of dopaminergic neurons in the substantia nigra [45]. In fact, PD-affected patients frequently show increased levels pro-inflammatory cytokines associated with the presence of bacterial inflammagens, such as *P. gingivalis* LPS and gingipain, in serum [24]. Moreover, these findings also reinforce the hypothesis of the existence of a neuroinflammatory oral-gut-brain axis behind neurodegenerative pathologies. Indeed, *P. gingivalis* is able to activate LLRK2 and enhance the expression α-synuclein in the gut, which can travel along the vagus nerve and provoke microglial overactivation and α-synuclein misfolding in the brain, leading to central neuroinflammation [45, 46]. In addition, inflammation in the gut caused by *P. gingivalis* may disrupt the gut barrier, allowing toxic molecules like LPS, among other pro-inflammatory mediators, into the bloodstream and lead to systemic inflammation that can affect the central nervous system [33]. Apart from that, the resulting circulating systemic pro-inflammatory cytokines, like IL-1 and IL-17 A, may also directly mediate neuroinflammation and consequent neurodegeneration [45]. Remarkably, high levels of IL-17 A are expressed during both periodontitis and peri-implantitis, locally and systemically [47, 48]. Therefore, it is possible to suggest that the presence of the same pro-inflammatory and osteolytic mediators present in PI-affected tissues, and at the same time, in the gut, serum and brain, definitely traces a sequence of inflammatory molecular events that could start from the mouth, go through the gut and systemic circulation, and end in neuroinflammatory events that lead to neuropathologic changes in the brain.

Elevated IL-6 levels have been frequently observed in peripheral blood of both AD and PD affected patients in different studies [49–51]. Distinct pro-inflammatory pathways in the brain commonly coincide with IL-6 signaling, which provokes metabolic alterations that lead to memory dysfunction during AD and motor dysfunction during PD [50, 51]. In this context, signs of hypothalamic/hippocampal inflammation and motor symptoms severity positively correlate with plasma IL-6 levels, and these IL-6 concentrations negatively correlate with cognitive performance in both AD and PD affected patients [50, 51]. In addition, the blockade of IL-6 signaling prevents microglial pro-inflammatory activation and neuroinflammation, and improves long-term memory performance, suggesting its active role during regulation of the neuroimmune network and memory associated metabolism [50, 52, 53]. However, locally produced IL-6 also plays several roles within brain physiological functions, by being able to signal through receptors commonly expressed on neurons, astrocytes, and microglia. For instance, IL-6 can modulate synaptic plasticity in neurons, gliotransmitter release in astrocytes, and promote microglia pro-inflammatory activity in case of injury or infection [54]. Additionally, IL-6 is vital for the differentiation of oligodendrocytes, the regeneration of peripheral nerves, acting as a neurotrophic factor [55]. Apart from that, IL-6-mediated microglial activation promotes Aβ clearance in mice hippocampus [56]. Therefore, the overlapping threshold levels of IL-6 corresponding to health or disease found among patients, animal and in vitro models, may suggest that the differences between a state of health or disease may rely on IL-6 differential signaling pathways, i.e., cis or trans, rather than the concentration of the cytokine [55, 57], thus drawing attention to the development of functional studies on IL-6 signaling role during neuroinflammatory pathologies.

Similarly, increased levels of TNF-α found in AD and PD affected patients’ cerebrospinal fluid and serum directly correlate with the diseases’ markers of severity, including cognitive decline [58–60]. In addition, TNF-α is major mediator of neurodegenerative neuroinflammation, being neurotoxic to oligodendrocytes, astrocytes, and neurons, while being considered neuroprotective in particular cases of excitotoxic injury in different parts of the brain [61]. Otherwise, its systemic inhibition has shown promising results by enhancing cognitive performance and decreased levels of cerebral Aβ during AD, and attenuating neurodegeneration and behavioral deficits during PD [61, 62], Both IL-6 and TNF-α mediate microglial pro-inflammatory activity, Aβ production, and dopaminergic neuron apoptosis, thus being considered major cytokines involved in AD and PD neuroinflammatory pathogenesis [61, 63]. However, the low-grade neuroinflammation detected in this model could not be associated to Aβ detection, probably due to immunohistochemistry low sensitivity, in comparison with other techniques like ELISA, and the molecular differences between wild type rats Aβ protein processing, which prevent the formation of the characteristic amyloid plaques and the pathological cleavage of detectable amounts of protein in the brain [Do Carmo 2013, Echeverria 2012]. Altogether, these results suggest that oral chronic inflammation triggered by dysbiosis, such as the cases of PI and periodontitis, may promote and, further aggravate, neuroinflammation in the brain, and compromise cognitive/motor functions, which are associated with the onset of neurodegenerative diseases like AD and PD.

Neuropathological conditions, including AD and PD, are outlined by the dysfunctional interplay between microglia and astrocytes activity [64, 65]. Indeed, the continuity of a glial-mediated deregulated neuroinflammation, including the major production of pro-inflammatory cytokines like IL-6 and TNF-α [66], is decisive for late stages of neurodegeneration [64, 67]. In the context of periodontal diseases, experimental periodontitis has led to higher immunodetection of IBA-1^+^ microglia at the cerebral cortex and the hippocampus, in comparison with control mice [68]. Otherwise, constant *P. gingivalis* oral application led to both increased IBA-1^+^ microglia and GFAP^+^ astrocyte detection in infected wild-type mice hippocampus [25]. Similarly, the use of LPS from *P. gingivalis* to induce periodontal inflammation also induced microgliosis and astrocytosis in the cerebral cortex [69], which altogether coincide with our results. Furthermore, it is presumed that the onset of periodontal diseases may prime microglia to an activated state and alter their cytokine profile, and predispose the brain to deregulated neuroinflammation and consequent neurodegenerative dementias, such as AD or PD [68, 70]. Thus, it is possible to suggest that the neuroinflammatory risk associated to peri-implantitis may also favor the susceptibility of the host to the onset of possible neuropathologies.

*P. gingivalis* is considered as a keystone pathogen, which despite its low relative abundance, is capable of remarkably influence polymicrobial synergy and favor the dysbiosis that leads to the osteodestructive inflammation characteristic of PI and periodontitis [71–73]. Interestingly, *P. gingivalis* may promote neuroinflammation and neurodegeneration by different mechanisms, either by directly activating microglia, Aβ production and α-synuclein misfolding in the brain or indirectly by pro-inflammatory mediators or virulence factors, like its LPS, released into systemic circulation and, subsequently, reaching the central nervous system and inducing neurotoxicity [74, 75]. In this context, one recent study demonstrated that experimental periodontitis induced by *P. gingivalis* provoked memory deficiency, led to changes in astrocytes morphology and increased Aβ 1–42 levels in the hippocampus of rats [74]. In fact, the severity of the AD model neurodegenerative signs appeared to be more evident in rats infected with encapsulated serotypes of *P. gingivalis*, which present a particular LPS configuration, compared with those infected with the non-encapsulated serotypes, thus suggesting that neuroinflammation associated to AD arises from the *P. gingivalis* strains able to elicit a more pronounced osteolytic inflammation [74]. Those findings corroborate results of one former in vitro analysis, which explored the inflammatory response triggered by *Aggregatibacter actinomycetemcomitans*, a periodontal pathobiont, in the hippocampus cells and cortical microglia of rats [76]. Specifically, LPS from *A. actinomycetemcomitans* induced pro-inflammatory cytokine production (i.e., IL-1b, IL-6, IL-17, and TNF-a) in microglia and hippocampal cells, and increased extracellular plaque (Aβ 1–42) production, thereby depicting the differential brain’s immune response triggered by its LPS [76]. On the other hand, another study using a PD model, showed that the inoculation of *P. gingivalis* led to a significant reduction of dopaminergic neurons and marked microglia activation [45]. The aforementioned findings further align with numerous previous in vivo studies that demonstrated the association between neuropathology and chronic oral infection and/or dysbiosis resulting from either repeated oral applications of *P. gingivalis* [77, 78], *P. gingivalis*-induced periodontitis [25, 79], the translocation of its LPS [80], or induced pro-inflammatory cytokines [27].

The discrepancies between the present results and previous experimental studies might be, at least partially, attributed to the fact that the present analysis employed LPS from *Escherichia coli*, which is not a typical microorganism of rat oral microbiota and features distinct virulence properties compared to those of *P. gingivalis* or *A. actinomycetemcomitans* [81], thus possibly compromising its translational application. However, *E. coli* LPS is also considered as a strong activator of microglial activity and has been frequently used in neuroinflammation AD and PD related studies [82–86]. In fact, *E. coli* LPS, colocalizing with Aβ 1–42 plaques, has been detected in brains from AD affected patients [87]. Similarly, increased detection of *E. coli* and intestinal permeability, correlating with α-synuclein, have been found in PD affected patients [88]. In this context, induced mono-colonization with *E. coli* in mice leads to α-synuclein pathology compatible with PD, in the gut and the brain [89]. Moreover, *E. coli* LPS has shown an enhanced capacity to induce microglial pro-inflammatory cytokine production, in comparison with *P. gingivalis* LPS, astrogliosis and α-synuclein dysfunction [86, 90, 91]. Thus, these results also reinforce the hypothesis that postulate how gut bacteria, like *E. coli*, and their products may also be associated to neuroinflammatory pathogenesis; for instance, LPS overexposure promotes gut dysbiosis and induces neuroinflammation through the peripheral enteric nervous system [92] or LPS may be carried by immune phagocytes directly into the brain [87]. Nevertheless, the single dose, via and concentration of LPS administration and the ligature remained in place used in the present study is probably insufficient to induce long-term neuropathologic changes in the rat brain, otherwise daily applications, intracerebral injections, and much higher concentrations are currently used in neuroinflammation, neurotoxicity and neuropathology rat and mice models [93–95].

Moreover, it was previously demonstrated that chronic prolonged exposure to risk factors might be necessary for the development of neurodegenerative changes in the brain, and a single dose of *P. gingivalis* application was shown to be insufficient to induce Aβ 1–42 production, whereas three doses a week for a month were needed for the induction of α-synuclein accumulation, in two different mice models [45, 68]. In fact, in a recent experimental study in a rat model, the total induction period of periodontitis was 6 weeks and the *P. gingivalis* injections were repeated twice, with a 7-day period in between [74]. Subsequently, it might be speculated that the lack of repeated applications of LPS during the progression period of PI in the present study might have further influenced our outcomes. Apart from that, it would be valid to consider the fact that tooth loss leads to the loss of periodontal neuronal connections, masticatory sensitivity and, consequently, it may diminish central nervous system activity [96], and these may not be restored by implant-supported treatment. Indeed, multiple studies associate tooth loss with cognitive decline in the elderly [97], however most of these studies do not explore the causes behind tooth loss, or merely discuss the mostly chronic inflammatory (periodontitis or caries) disease that led to tooth loss, and from which surgical extraction for ‘other reasons’ are the least common causes [96]. For instance, Oue et al. [98] found no differences regarding Aβ deposit, hippocampal neuron number, learning or memories abilities between extracted teeth group and no extraction after 4 months in a transgenic Alzheimer’s disease predisposed animal model. Therefore, tooth extraction per se may not significantly contribute to oral chronic inflammation associated to neuropathology.

Ultimately, to the authors’ best knowledge, this is the first study employing a PI model in rats to assess its possible link with neuroinflammatory changes associated with neurodegenerative disorders, such as AD or PD. However, the limited number of available animals/brain samples, the sole use of immunohistochemistry without any other complementary molecular assessment techniques, the use of LPS not derived from an oral pathogen, the absence of cognition/motor skills-associated behavior analysis, and the performance of an implant surface decontamination procedure limit the extrapolation of these preliminary results. In this context, taking our findings into account, future studies should consider the detection of oral microbial products, such as LPS and toxins from known peri-implantitis associated bacteria, in plasma, cerebrospinal fluid and brain, in order to confirm a via of microbial translocation through the blood-brain barrier caused by peri-implantitis. In this sense, the detection of systemically diffused pro-inflammatory mediators in plasma and distal organs, by means of mass spectrometry, Western Blot and/or ELISA, could better suggest the role of low-grade systemic inflammation in neuroinflammation provoked by peri-implantitis [99].

## Conclusions

Within the limitations of the present experimental exploratory study, our analysis showed that chronic PI lesions associated with the increased detection of IL-6 and TNF-α, and the microgliosis and astrocytosis markers GFAP and IBA-1, thus showing neuroinflammatory signs associated with neurodegenerative disorders, such as AD or PD.

## Data Availability

Additional data will be provided on reasonable request.
