# Correction: Experimental peri-implantitis induces neuroinflammation: An exploratory study in rats

**DOI:** 10.1186/s12903-025-05736-6

**Published:** 2025-04-03

**Authors:** Emilio A. Cafferata, Ausra Ramanauskaite, Astrid Cuypers, Karina Obreja, Eva Dohle, Shahram Ghanaati, Frank Schwarz

**Affiliations:** 1 Department of Oral Surgery and Implantology, Goethe University, Carolinum, Frankfurt Am Main, Germany; 2 Oral Peri-Implant Research Group, School of Dentistry, Universidad Científica del Sur, Lima, Perú; 3 Frankfurt Oral Regenerative Medicine (FORM-Lab), Clinic for Maxillofacial and Plastic Surgery, Goethe University, Frankfurt Am Main, Germany

**Correction** **:** **BMC Oral Health 24, 1238 (2024)**

**https://doi.org/10.1186/s12903-024-04995-z**

In this article [1], part of the Fig. 3, Fig. 7 and Fig. 8 are missing, specifically panels Fig. 3b-c-d, Fig. 7g-h, and Fig. 8g-h, which depict the relative quantification of pyknotic neurons, IBA-1^+^ and GFAP^+^ cells, respectively. The Figs. 3, 7 and 8 should have appeared as shown below.

**Fig. 3:**
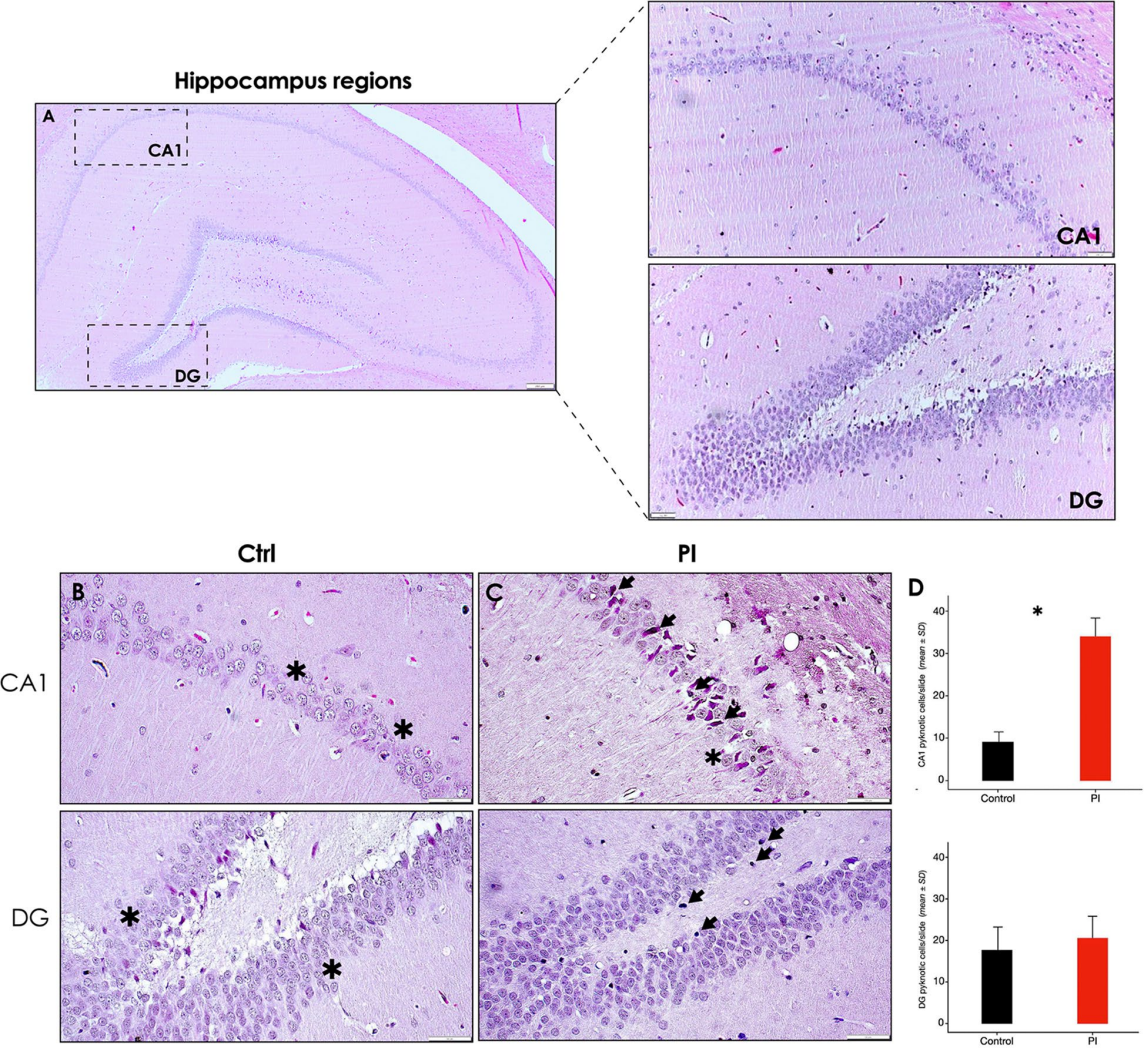
Hippocampus H&E staining showing cellular morphological alterations following experimental PI. **A** Representative image showing the hippocampus regions -CA1, CA2, C3, CA4 and DG-, the regions of interest CA1 and DG are delimited by dashed squares [5x]; Right: Representative imagesof the CA1 (superior) and DG (inferior) hippocampus regions of the control group [10x]. **B** and **C** Representative images of the CA1 (superior) and DG(inferior) hippocampus regions of the control and peri-implantitis group, showing morphologically altered neurons and glial cells [40x]. **D** Relative quantificationof pyknotic cells in the CA1 and DG regions of the hippocampus of rats in control and PI groups. Data are represented as mean ± SD pyknoticcells from 10 random slides per sample. *p < 0.05.CA: Cornu ammonis; DG: dentate gyrus; PI: peri-implantitis group; CTRL: control group; ↘: pyknotic pyramidal neuron; *: normal neuron. Figure scales correspond to: superior [200 μm], superior right [100 μm] and inferior [50 μm] figures

**Fig. 7:**
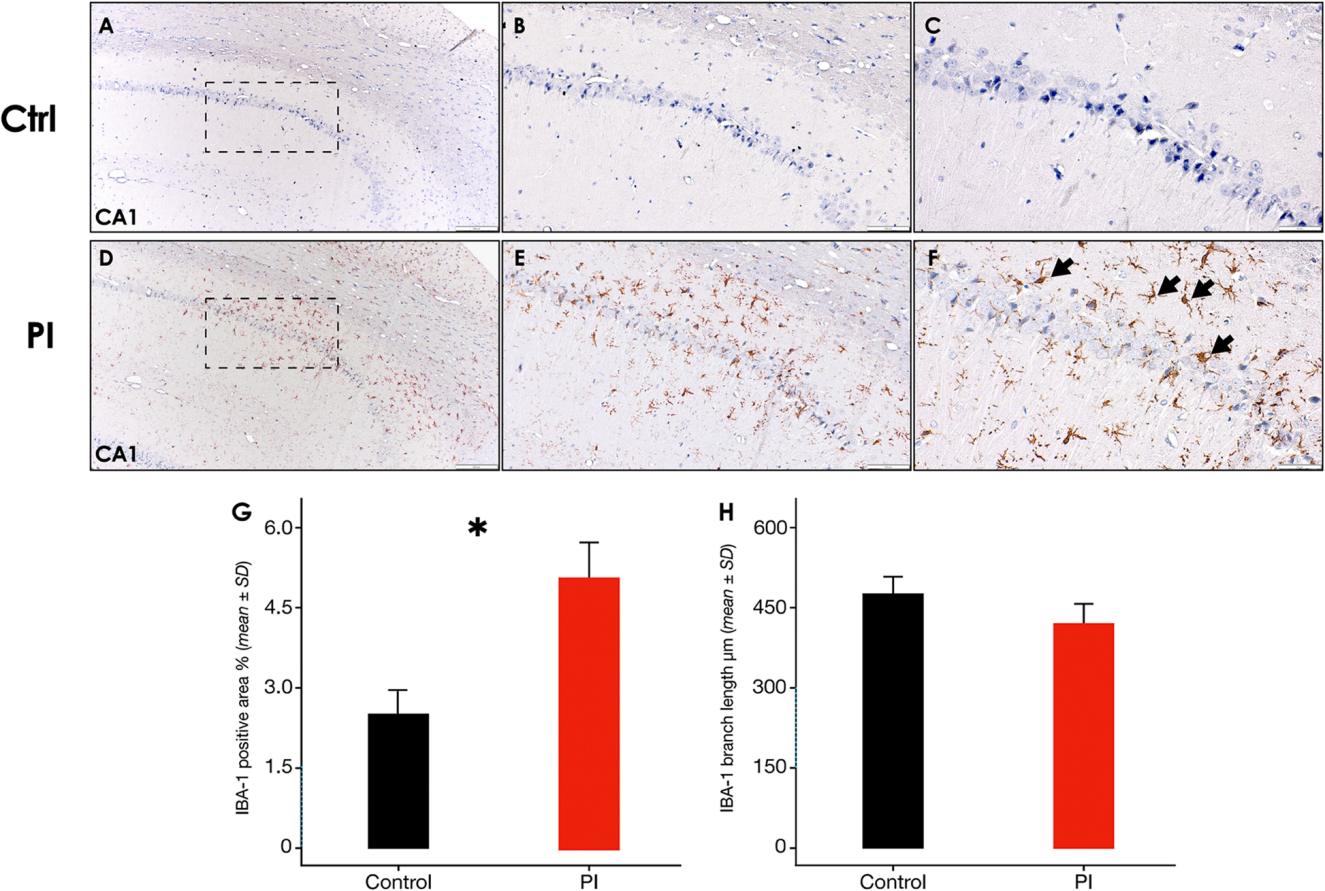
Hippocampus IBA-1 immunostaining following experimental PI. **A** Representative hippocampus image of the control group, showing the CA1 region, a zoom augmented area is delimited by a dashed square [10x], **B** [20x] and **C** [40x]. **D** Representative hippocampus image of the peri-implantitis group, showing the CA1 region, a zoom augmented area is delimited by a dashed square [10x], **E** [20x] and **F** [40x]. **G** IBA-1^+^ surface coverage percentage and H) IBA-1^+^ Microglia branch length in the CA1 region of the hippocampus of rats in control and PI groups. Data are represented as mean ± SD of the percentage of positively stained area from 10 random slides per sample, or mean ± SD of the branches length divided by the number of cells per slide from 10 random slides per sample. *p < 0.05. PI: peri-implantitis group; CTRL: control group; ↘: IBA-1 positive cell. Figure scales correspond to:left [200 μm], middle [100 μm] and right [50 μm] figures

**Fig. 8:**
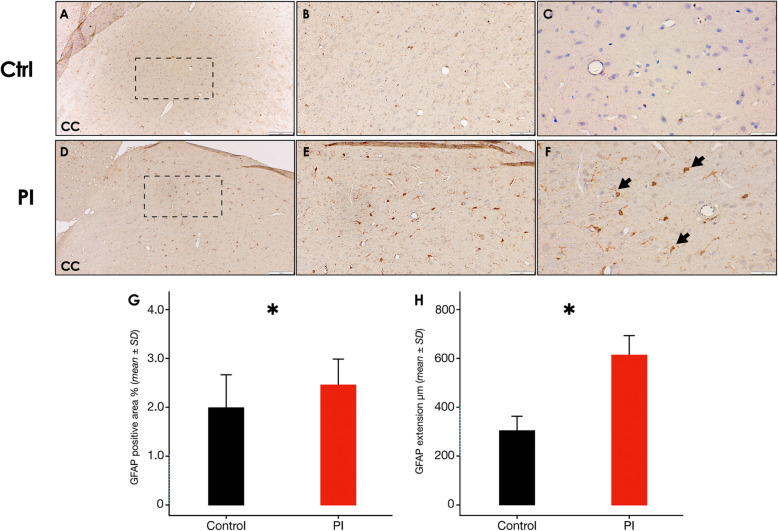
Cerebral cortex GFAP immunostaining following experimental PI. Representative image of the control group showing the cerebral cortex,a zoom augmented area is delimited by a dashed square [10x], **B** [20x] and **C** [40x]. **D** Representative image of the peri-implantitis group showing the cerebral cortex, a zoom augmented area is delimited by a dashed square [10x], **E** [20x] and **F** [40x]. **G** GFAP^+^ surface coverage percentage in the cerebral cortex of rats in control and PI groups. Data are represented as mean ± SD of the percentage of positively stained area from 10 random slides per sample. *p < 0.05. PI: peri-implantitis group; CTRL: control group; ↘: GFAP positive cell. Figure scales correspond to: left [200 μm], middle [100 μm]and right [50 μm] figures

The original article has been corrected.
